# Case Commentary: Overcoming intrinsic resistance—the successful use of aztreonam and avibactam for *Stenotrophomonas maltophilia* meningitis

**DOI:** 10.1128/aac.00640-25

**Published:** 2025-07-09

**Authors:** Alice J. Hsu, Pranita D. Tamma

**Affiliations:** 1 Department of Pharmacy, The Johns Hopkins Hospital 588543, Baltimore, Maryland, USA; 2 Department of Pediatrics, Division of Pediatric Infectious Diseases, The Johns Hopkins University School of Medicine 1500, Baltimore, Maryland, USA; Houston Methodist Hospital and Weill Cornell Medical College, Houston, Texas, USA

**Keywords:** *Stenotrophomonas maltophilia*, aztreonam-avibactam, cefiderocol, meningitis

## Abstract

Wong-So and colleagues describe a case of post-neurosurgical *Stenotrophomonas maltophilia* meningitis successfully managed with ceftazidime-avibactam and aztreonam. While aztreonam is stable against L1 metallo-β-lactamase-mediated hydrolysis, it remains susceptible to L2 serine β-lactamases, which are effectively inhibited by avibactam, restoring aztreonam’s bactericidal activity. Simultaneous peak and trough levels in cerebrospinal fluid (CSF) and plasma demonstrated favorable CSF-to-plasma ratios for both aztreonam and avibactam. This case highlights aztreonam-avibactam as a rational therapeutic strategy for *S. maltophilia* meningitis.

## COMMENTARY

We commend Wong-So and colleagues for their successful management of post-neurosurgical *Stenotrophomonas maltophilia* meningitis in an adult patient using the antibiotic combination of ceftazidime-avibactam and aztreonam (1). This regimen functionally replicates the activity of aztreonam-avibactam, a β-lactam/β-lactamase inhibitor combination approved by the European Medicines Agency and the United States Food and Drug Administration (FDA) (2). The authors conducted meticulous pharmacokinetic assessments, quantifying simultaneous peak and trough concentrations of aztreonam and avibactam in both cerebrospinal fluid (CSF) and plasma to derive CSF-to-plasma ratios—yielding robust data on central nervous system (CNS) penetration throughout the 14 day treatment course. Notably, no other agents with activity against *S. maltophilia* were administered, underscoring that the patient’s favorable clinical outcome can be attributed to this combination. These findings support the therapeutic potential of aztreonam in combination with avibactam for CNS infections and contribute to the growing body of evidence endorsing aztreonam-avibactam for the treatment of *S. maltophilia* infections.

*S. maltophilia* is an opportunistic pathogen that preferentially colonizes vulnerable hosts with disrupted microbiota (3). As in the present case, the majority of individuals with *S. maltophilia* infection have a history of substantial prior antibiotic exposure—underscoring that the most critical modifiable risk factor for prevention is the judicious use of antimicrobials (4).

The *S. maltophilia* isolate in this case exhibited a minimum inhibitory concentration (MIC) of 4/4 µg/mL for aztreonam-avibactam. Susceptibility breakpoints for aztreonam-avibactam against *S. maltophilia* have not been established by the European Committee on Antimicrobial Susceptibility Testing (EUCAST), the Clinical and Laboratory Standards Institute (CLSI), or the FDA. A recent analysis of 1,400 *S*. *maltophilia* isolates collected in the United States between 2019 and 2023 reported an MIC₉₀ of 4/4 µg/mL for aztreonam-avibactam (5)—a value that aligns with the current EUCAST, CLSI, and FDA susceptibility breakpoint for Enterobacterales.

Both *in vitro* and clinical data evaluating the efficacy of aztreonam-avibactam against *S. maltophilia* remain limited. In time-kill studies of six *S*. *maltophilia* isolates non-susceptible to levofloxacin and/or trimethoprim-sulfamethoxazole, the addition of avibactam to aztreonam resulted in ≥2-log₁₀ CFU/mL reductions at 24 hours in 83% of isolates (6). The pharmacokinetic/pharmacodynamic (PK/PD) target necessary to achieve a ≥1 log bacterial kill has yet to be defined. Clinical experience is similarly scarce and limited to case reports (7–10). In a clinical trial comparing aztreonam-avibactam with alternative therapy for metallo-β-lactamase-producing infections (NCT03580044), three patients with *S. maltophilia* were randomized to receive aztreonam-avibactam; outcomes included one clinical response, one indeterminate response, and one treatment failure (private communication, AbbVie).

Despite notable gaps in both preclinical and clinical data, there are compelling mechanistic and *in vitro* findings that support the potential role of aztreonam-avibactam for the treatment of *S. maltophilia* infections. Most conventional β-lactams are rendered ineffective by the inducible L1 metallo-β-lactamase produced by *S. maltophilia* isolates. Aztreonam, however, remains uniquely stable against L1-mediated hydrolysis due to its ability to occupy the shallow groove of the L1 enzyme, but given that it is a small, monocyclic compound, it is improperly positioned for hydrolysis (11). Nonetheless, aztreonam is susceptible to degradation by co-produced L2 serine β-lactamases. Avibactam effectively inhibits these L2 enzymes, thereby protecting aztreonam and allowing it to bind to its target, penicillin-binding protein 3, and exert bactericidal activity against *S. maltophilia*.

We respectfully disagree with the authors’ dismissal of cefiderocol as a therapeutic option for *S. maltophilia* infections. Cefiderocol demonstrates potent *in vitro* activity against *S. maltophilia*, with susceptibility rates approaching 100% (12). In a neutropenic rabbit model of *S. maltophilia* pneumonia, cefiderocol treatment was associated with significantly reduced mortality compared to trimethoprim-sulfamethoxazole (13). Admittedly, as with aztreonam-avibactam, clinical evidence supporting the use of cefiderocol for *S. maltophilia* infections remains largely confined to case reports (14–18). In a clinical trial comparing cefiderocol to alternative therapies for carbapenem-resistant infections, all five patients with *S. maltophilia* infections were randomized to the cefiderocol arm; only one survived (19). Nonetheless, we believe the cumulative data—albeit predominantly from *in vitro* studies, animal models, and case reports—support cefiderocol as a promising therapeutic option for *S. maltophilia,* making it at least comparable to aztreonam-avibactam. Furthermore, cefiderocol appears to achieve therapeutic concentrations in the CSF, with over 80% of published case reports describing clinical cure in gram-negative meningitis treated with this agent (20).

In addition to reinforcing the potential efficacy of aztreonam-avibactam for *S. maltophilia* infections, a key contribution of the present case is the demonstration that this combination achieves therapeutically relevant CSF concentrations. The patient’s peak CSF-to-plasma ratios of aztreonam ranged from 13% to 26% early in the treatment course, declining to <10% by its conclusion. Similarly, trough CSF-to-plasma ratios for aztreonam were ~40% during the initial days of therapy, decreasing to ~17% by day 7. Avibactam pharmacokinetics in this case demonstrated peak CSF-to-plasma ratios of 9%–13% during the first week, decreasing to 7% by day 13; trough ratios ranged from 25% to 31% early in therapy and declined to ~16% by day 7. Notably, it is not uncommon for CSF trough concentrations to exceed CSF peak concentrations due to the slower rate of CSF clearance compared to plasma (21).

To contextualize these findings, CSF penetration during meningeal inflammation has been reported to be 20% for penicillins, 15% for cephalosporins, and 30% for carbapenems (22). In contrast, penetration declines in the absence of inflammation—typically observed later in the course of therapy—to 2%, 10%, and 20%, respectively (22). Taken together, the CSF concentrations of both aztreonam and avibactam appear comparable to those achieved by other β-lactams with established efficacy in the treatment of gram-negative meningitis, such as ceftazidime (23).

In summary, the case presented by Wong-So and colleagues provides important preliminary evidence supporting the use of aztreonam-avibactam for the treatment of *S. maltophilia*, including CNS infections. Further studies are warranted to establish susceptibility breakpoints for aztreonam-avibactam against *S. maltophilia* and to define the PK/PD targets necessary to optimize dosing strategies—such as frequency of administration (e.g., every 6 versus every 8 hours) and infusion duration (e.g., 30 minute versus 3 hour infusions)—in the management of CNS infections.
